# Knowledge mapping of autophagy in chronic obstructive pulmonary disease from 2004 to 2024: a bibliometric analysis

**DOI:** 10.3389/fmed.2025.1514686

**Published:** 2025-02-24

**Authors:** Yating Zhang, Anqi Li, Sumei Xu, Haoge Liu

**Affiliations:** ^1^Qinggang Town Health Center, Yuhuan Second People's Hospital Health Community Group Qinggang Branch, Taizhou, China; ^2^Guang’anmen Hospital, China Academy of Chinese Medical Sciences, Beijing, China; ^3^The First Affiliated Hospital of Zhejiang Chinese Medical University (Zhejiang Provincial Hospital of Chinese Medicine), Hangzhou, China

**Keywords:** autophagy, chronic obstructive pulmonary disease (COPD), bibliometric analysis, CiteSpace, VOSviewer

## Abstract

**Background:**

Chronic obstructive pulmonary disease (COPD) has become a major global healthcare issue due to its high prevalence and mortality rates. Increasing evidence suggests that autophagy plays a role in the development of COPD, yet there is a lack of bibliometric analyses on literature related to autophagy and COPD. Therefore, this study aims to summarize the current research status, research direction, and research hotspots in the literature related to COPD and autophagy.

**Methods:**

A search was conducted on the Web of Science Core Collection for literature related to COPD and autophagy from October 2004 to October 2024. Subsequently, bibliometric and visualization analyses were performed on the included publications using R software, CiteSpace, and VOSviewer.

**Results:**

A total of 481 published articles across 229 journals related to COPD and autophagy were included. During the study period, there was a trend of continuous growth in both the annual number of publications and citations in this field. The United States had the highest centrality, while China was the most productive country. Major research institutions included Maastricht University, Harvard University, and Jikei University. The American Journal of Physiology-Lung Cellular and Molecular Physiology, International Journal of Chronic Obstructive Pulmonary Disease, and Autophagy are the most influential journals in this field. The author with the most published papers is Araya J, while Choi AMK is the most influential author. Frequently appearing keywords include “oxidative stress,” “obstructive pulmonary disease,” “aging,” “apoptosis,” and “mitophagy.”

**Conclusion:**

This bibliometric study helps researchers quickly understand the research overview of autophagy and COPD, thus providing new insights and directions for future research in this field.

## 1 Introduction

Chronic obstructive pulmonary disease (COPD) is a common, preventable, and treatable disease characterized by persistent airflow limitation, usually progressive. This limitation is linked to an enhanced chronic inflammatory response in the lungs to harmful particles or gases, with risk factors including cigarette smoke, air pollution, and genetics (1, 2). The pathophysiology involves narrowing small airways and destruction of alveoli, leading to an imbalance in lung ventilation and perfusion (3). Patients often present with persistent dyspnea, chronic cough, and sputum production (4). Acute exacerbations and comorbidities can worsen the disease. According to the World Health Organization, COPD affects approximately 300 million people worldwide and causes about 3 million deaths in 2019, making it the third leading cause of death globally (5). Although traditionally more common in men, prevalence in women is rising due to increased tobacco use and exposure to indoor pollutants (6). The chronic nature and severity of COPD highlight the urgent need for research to reduce its healthcare burden. Despite advancements, the underlying mechanisms remain not fully clarified. The complex pathogenesis likely involves numerous pathways, including chronic inflammation, oxidative stress, protease-antiprotease imbalance, airway remodeling, and cellular processes like autophagy (7–9). Understanding these mechanisms is vital for developing targeted therapies to effectively manage or modify the disease, improving quality of life and survival rates for patients.

Autophagy is a crucial cellular process that degrades and recycles damaged organelles and proteins, maintaining homeostasis and aiding survival in stress conditions (7, 10). This process is regulated by key proteins and pathways, including mTOR and AMPK, which control autophagosome formation (11). The autophagosome, a double-membrane vesicle, captures cellular waste with the help of proteins like LC3 and p62. It then fuses with a lysosome to form an autolysosome, where the contents are degraded and recycled (12, 13). In COPD, autophagy clears damaged cells and reduces oxidative stress, but dysregulation can worsen the condition. Thus, autophagy represents a potential therapeutic target for disease management by restoring cellular balance.

Bibliometrics is a scientific analytical method that quantitatively evaluates research trends, academic impact, and research networks through the analysis of academic literature. It utilizes quantitative tools and indicators such as publishing volume, citation counts, impact factors, h-index, and collaboration network mapping to reveal development patterns and trends in specific academic fields (14). Bibliometric analysis typically includes the analysis of time series data, evaluation of the influence of journals and authors, identification of research hotspots, and visualization of scientific collaboration patterns (15). These analyses not only help assess the academic impact and dynamics of research areas but also provide data support for research decision-making, playing a significant role in the formulation of research policies and resource allocation (16). Despite the growing attention to autophagy and its roles in various diseases, there is still a lack of bibliometric analysis focusing specifically on autophagy in the field of COPD. The purpose of this study is to conduct a bibliometric analysis of the existing literature on autophagy in COPD to uncover research trends, key studies, and potential areas for future research directions.

## 2 Materials and methods

### 2.1 Search strategy

The literature search was conducted using the Web of Science Core Collection (WoSCC) database. The search terms employed were as follows: TS = (“chronic obstructive pulmonary disease” OR “COPD” OR “chronic airflow obstruction” OR “chronic obstructive lung disease”) AND TS = (“autophagy” OR “macroautophagy” OR “microautophagy” OR “autophagosome” OR “lysosome” OR “autophagic flux” OR “mitophagy” OR “lipophagy” OR “LC3” OR “p62”).

### 2.2 Data screening

#### 2.2.1 Inclusion criteria

1. Literature related to autophagy and acute myeloid leukemia.
2. Literature published in English.
3. Literature types include clinical trial studies, *in vitro* experimental studies, *in vivo* experimental studies, public database analysis studies, reviews, etc.
4. Literature with complete bibliographic information (including title, country, author, keywords, source).

#### 2.2.2 Exclusion criteria

1. Conference papers, newspapers, patents, achievements, health and popular science literature, etc.
2. Duplicate publications.

### 2.3 Data analysis

In this study, data analysis was conducted using a suite of specialized software tools, including VOSviewer, CiteSpace, and R. The introduction of each tool and its role in bibliometrics are as follows:

VOSviewer is a software tool designed for constructing and visualizing bibliometric networks. It is widely used in bibliometric analysis to create maps of co-authorship, keyword clustering, and citation networks. Through these visualizations, researchers can identify academic collaborations, focal research themes, and citation relationships among scientific outputs.

CiteSpace is a tool focused on visualizing and analyzing emerging trends and patterns in scientific literature. In bibliometrics, it is used to perform temporal analysis, helping to detect critical turning points in research areas, identify highly cited key documents, and discover emerging research topics. CiteSpace’s visualizations aid researchers in understanding the developmental trajectory and dynamic characteristics of the field.

R is a powerful tool for statistical analysis and data visualization, used in bibliometric research to perform complex data analyses and creative graph creation. With R, researchers can conduct in-depth statistical examinations and dynamically present results, thereby enriching the depth and breadth of literature analysis.

## 3 Results

### 3.1 Quantitative analysis of publication

**Figure 1:**
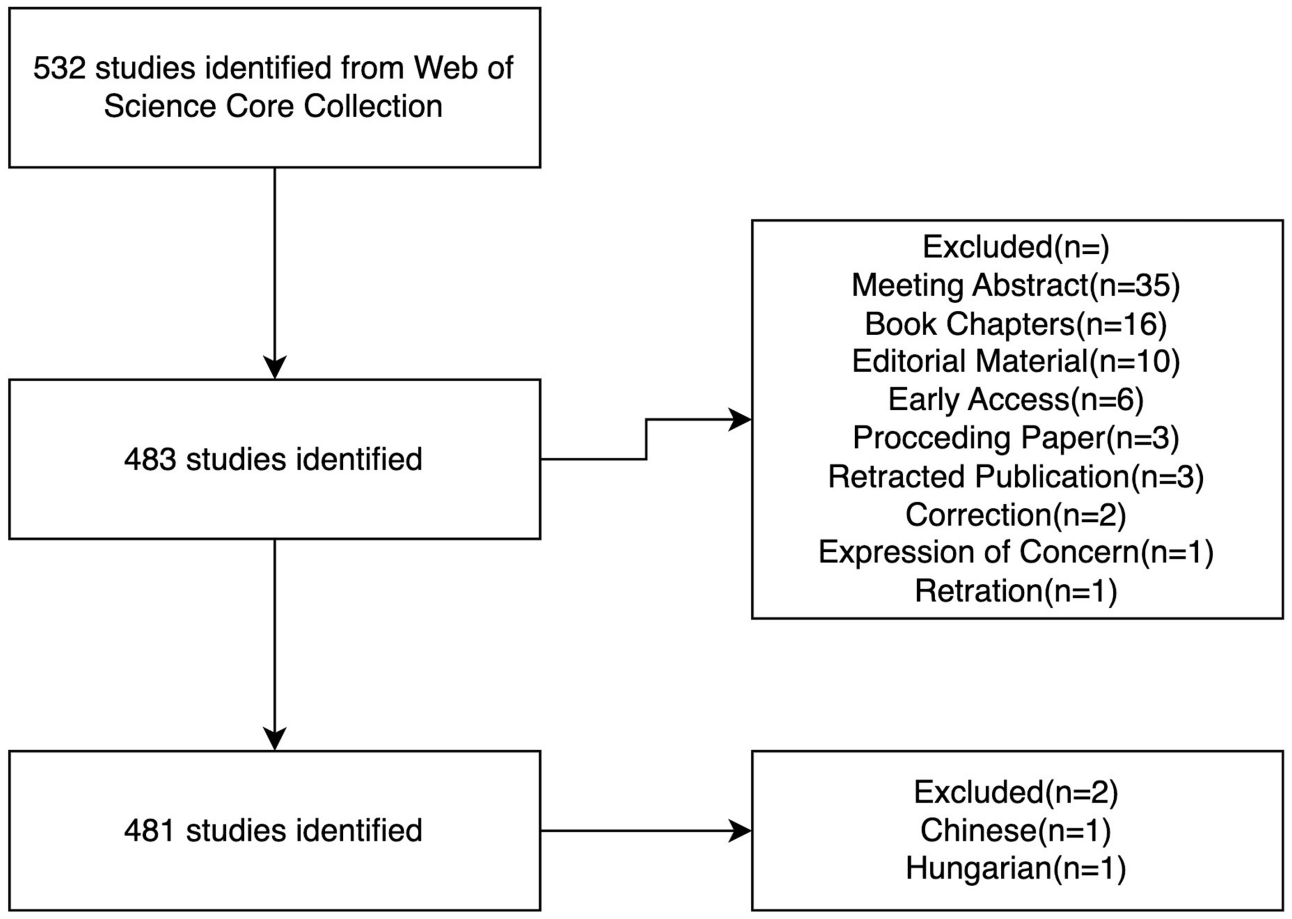
Flowchart for bibliometrics analysis.

Through our comprehensive search strategy, we identified a substantial body of research on autophagy in COPD, including 348 research articles and 133 review articles (Figure 1). Overall, a total of 2,414 authors from 39 countries published articles related to autophagy in COPD in 229 journals worldwide. The included literature had a total H-index of 67, and the average number of citations per article was 55.34, with three articles having more than 400 citations.

**Figure 2:**
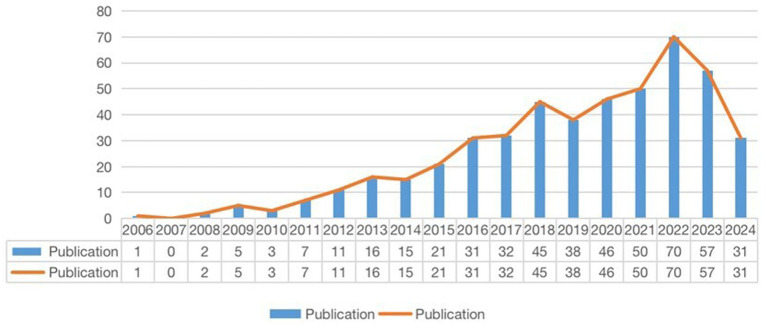
Annual output of research of autophagy in COPD.

As illustrated in Figure 2, the volume of these publications has consistently increased over the past 18 years, indicating growing interest and output in the study of autophagy’s role in COPD. During the initial phase from 2006 to 2011, the number of publications in this field remained relatively modest. The annual publication count did not exceed 10, suggesting that this period represented the early stage of research on autophagy in COPD. The limited number of publications during this time suggests that the relevant theories had not yet been comprehensively validated. However, over time, there has been a gradual increase in research on autophagy within the context of COPD, reflecting a growing interest in uncovering the complexities of COPD and its association with autophagy.

From 2012 to 2022, during the second phase, there was a substantial increase in the number of publications in this field, highlighting a significant surge in both interest and research activity among academics regarding autophagy in COPD. As the volume of publications grew, the scope of research findings expanded considerably. However, since 2023, in the third phase, there has been a gradual decline in the number of publications, suggesting that this area of research is no longer a major focus among researchers. Overall, these observations indicate that research on autophagy in COPD has evolved into a rapidly developing field with considerable potential.

### 3.2 Country and institutional analysis

**Figure 3:**
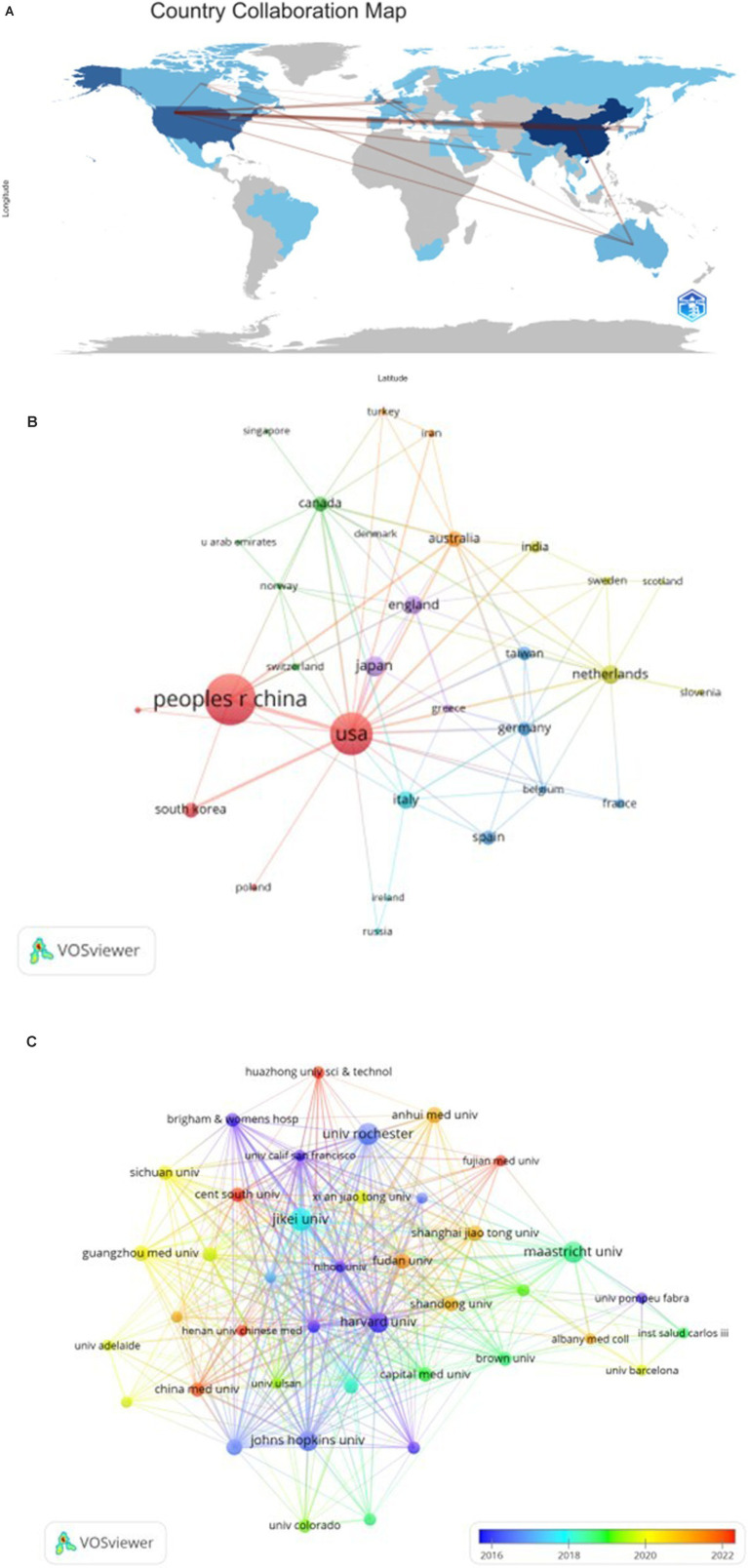
The geographical distribution **(A)** and visualization of countries **(B)** on the research of autophagy in COPD. The visualization of institutions **(C)** on the research of autophagy in COPD.

To assess global contributions to this research field, a detailed examination of the top 10 contributing countries reveals their distribution across Asia, North America, and Europe, with a predominant presence in Asia (*n* = 3) and Europe (*n* = 5), as shown in Table 1. Notably, China leads in publication output (*n* = 192, 39.9%), followed by the United States (*n* = 102, 21.2%), Japan (*n* = 27, 5.6%), and the Netherlands (*n* = 22, 4.6%). Collaborative publications between China and the United States account for more than half of the total output (54.5%). To comprehensively evaluate international collaboration, we filtered data from countries with two or more publications, yielding information from 24 countries. Figure 3 illustrates the dynamic collaboration among these countries, highlighting China’s active partnerships with the United States and Australia, while the United States shows a preference for collaboration with Japan and Korea.

**Table 1:** Top 10 countries and institutions on the research of autophagy in COPD.

Rank	Country	Counts	Institution	Counts
1	China	192	Maastricht University	47
2	USA	102	Harvard University	39
3	Japan	27	Jikei University	33
4	Netherlands	22	Maastricht University Medical Center (MUMC)	27
5	United Kingdom	18	Brigham and Women’s Hospital	25
6	Italy	17	Central South University	25
7	Spain	14	Johns Hopkins University	24
8	Korea	12	Henan University of Traditional Chinese Medicine	23
9	Australia	10	Harvard Medical School	21
10	Canada	10	China Medical University Taiwan	19

Upon review, it is evident that the top 10 institutions contributing to this field are spread across four countries, with a majority based in the United States. Among the top 10 institutions, five have emerged as the most prolific contributors: Maastricht University (*n* = 47), Harvard University (*n* = 39), Jikei University (*n* = 33), Maastricht University Medical Center (*n* = 27), and Brigham and Women’s Hospital (*n* = 25). Notably, among the top 10 institutions, six are located in the United States and three are in China, highlighting the significant presence of these two countries in the field. To further investigate, we visualized data from 40 institutions, each meeting a minimum publication threshold of 5. Figure 3C effectively illustrates this network, revealing intriguing patterns of collaboration.

### 3.3 Journal and cocited journals

The field of COPD autophagy research spans a broad spectrum, encompassing publications from 229 journals across various disciplines. These journals play a crucial role in disseminating knowledge in related fields. The frontrunner, the American Journal of Physiology-Lung Cellular and Molecular Physiology, has published the highest number of papers on this topic, totaling 20 articles. Following closely is the International Journal of Chronic Obstructive Pulmonary Disease with 18 papers, Autophagy with 11, and the American Journal of Respiratory Cell and Molecular Biology with 10 publications. In terms of impact factor, Autophagy ranks highest among the top 15 journals, boasting an impressive IF of 14.6 (Q1). Next in line is Free Radical Biology and Medicine, with an IF of 7.1 (Q2; Table 2).

**Table 2:** Top 10 journals and cocited journals for research of autophagy in COPD.

Rank	Journal	Count	IF	Q	Cocited journal	Cocitation	IF	Q
1	American Journal of Physiology-Lung Cellular and Molecular Physiology	20	3.6	Q1	American Journal of Respiratory and Critical Care Medicine	1,413	19.3	Q1
2	International Journal of Chronic Obstructive Pulmonary Disease	18	2.7	Q2	American Journal of Physiology-Lung Cellular and Molecular Physiology	985	3.6	Q1
3	Autophagy	11	14.6	Q1	Autophagy	964	14.6	Q1
4	American Journal of Respiratory Cell and Molecular Biology	10	5.9	Q1	American Journal of Respiratory Cell and Molecular Biology	853	5.9	Q1
5	Frontiers in Immunology	9	5.7	Q1	Plos One	845	2.9	Q1
6	Frontiers in Pharmacology	9	4.4	Q1	European Respiratory Journal	795	16.6	Q1
7	Plos One	9	2.9	Q1	Journal of Biological Chemistry	772	4	Q2
8	Scientific Reports	9	3.8	Q1	Journal of Clinical Investigation	666	13.3	Q1
9	Cells	8	5.1	Q2	Proceedings of the National Academy of Sciences of the United States of America	659	9.4	Q1
10	International Journal of Molecular Sciences	8	0	Q1	Cell	586	45.5	Q1

**Figure 4:**
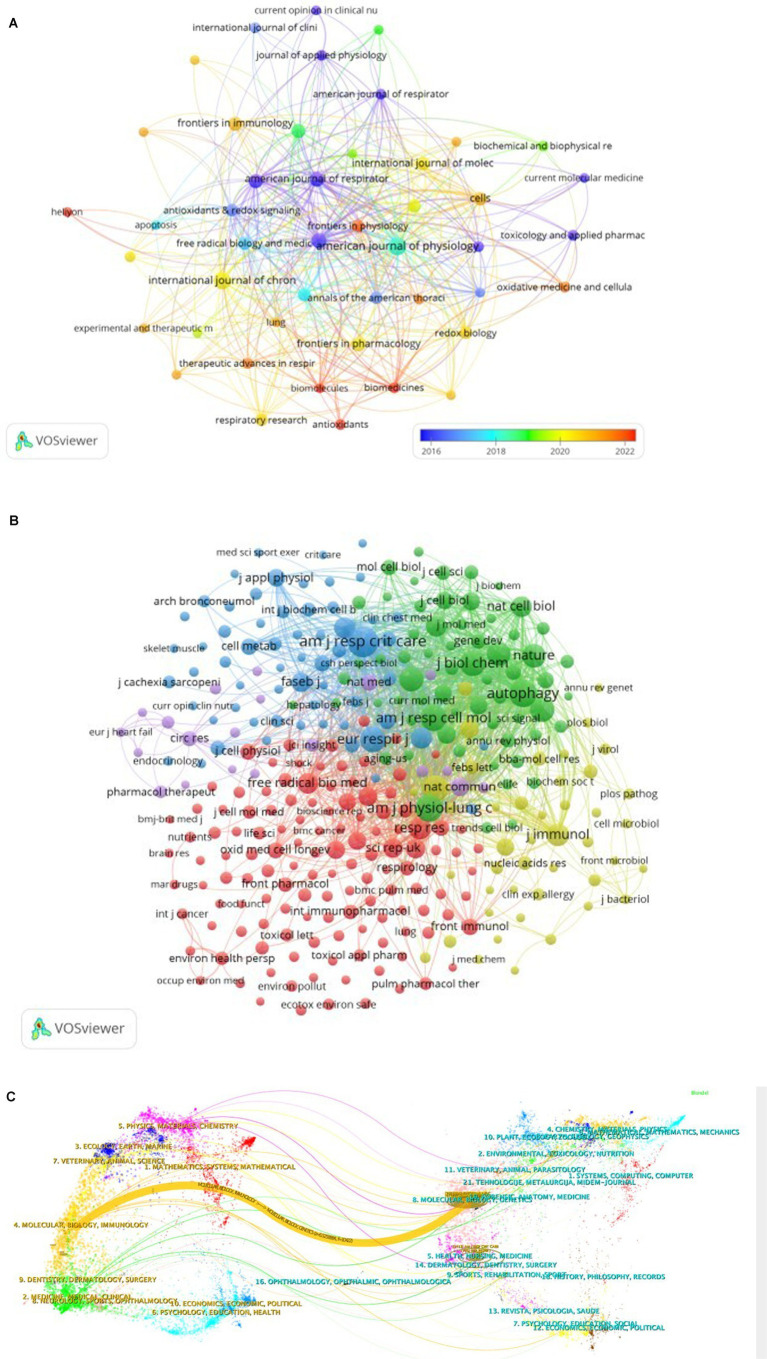
The visualization of journals **(A)** and cocited journals **(B)** on the research of autophagy in COPD. The dual-map overlay **(C)** of journals on the research of autophagy in COPD.

To provide a comprehensive overview of the relationships and citations among these journals, we employed a systematic screening method with a minimum publication threshold of three journals. A total of 46 journals were selected, and the journal network generated from these publications, as shown in Figure 4A, offers valuable insights into the interactions between them. Notably, the American Journal of Physiology-Lung Cellular and Molecular Physiology demonstrates active citation relationships with journals such as Autophagy, International Journal of Chronic Obstructive Pulmonary Disease, and Plos one, among others.

Co-citation between two articles indicates that both are referenced in the bibliography of a third article. Table 2 presents the co-citation data for the top 10 journals within this field of study. Notably, three journals have accumulated over 900 co-citations, with Am J Resp Crit Care leading the way (1,413), followed by Am J Physiol-Lung C (985), Autophagy (964), and Am J Resp Cell Mol (853). Additionally, when considering impact factors, Nature ranks highest (IF = 50.5), followed closely by Cell (IF = 45.5). To provide a comprehensive depiction of reverse citation relationships among journals, we applied a filter to select journals with a minimum co-citation count of 20 to be included in the mixed network (Figure 4B). As shown in Figure 4B, Am J Resp Crit Care demonstrates a strong positive relationship with journals such as Autophagy and J Biol Chem, highlighting active management and cross-referencing within the field.

The application of CiteSpace has considerably simplified the process of creating a dual-map overlay, which serves as a valuable instrument to delve into the disciplinary associations within the research landscape. Within this dual-map overlay, the left and right sections correspond to the journals that are citing and cited, respectively. These sections are interconnected by waves of citations. The citing side can be considered the forefront of ongoing research, whereas the cited side represents the fundamental knowledge upon which this research is built (Figure 4C).

In this analysis, we can observe the most significant and influential areas of study, prominently featuring disciplines such as “Molecular Biology” and “Immunology,” “Veterinary” and “Animal Science,” “Medicine,” “Medical,” “Clinical,” as well as “Physics,” “Materials,” and “Chemistry.”

The majority of citations lead toward two central fields: “Molecular Biology” and “Genetics,” and “Health,” “Nursing,” “Medicine.” Figure 4C provides a visual representation, with the orange path indicating the primary trajectory of citations and highlighting the predominant flow of scholarly influence.

### 3.4 Authors and cocited authors

**Figure 5:**
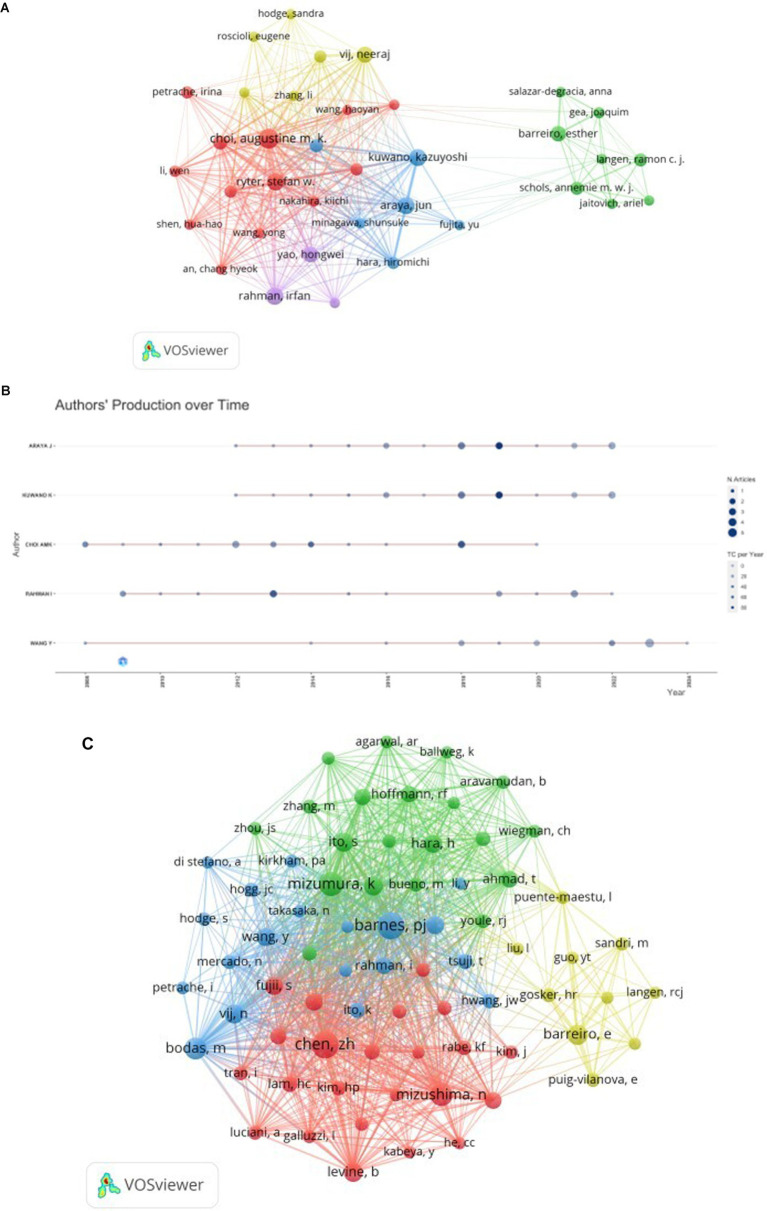
The visualization of authors **(A)** and cocited authors **(B)** on the research of autophagy in COPD. The node and line color represented the cluster it belonged to COPD.Authors’ production over time **(C)** on the research of autophagy in COPD.

A comprehensive analysis revealed that 2,414 authors contributed to the included literature. Table 3 presents the top 10 authors and cocited authors on the research of autophagy in COPD.The most prolific authors in this field were Araya J (*n* = 19), Kuwano K (*n* = 18), Choi AMK (*n* = 18), Rahman I (*n* = 16), and Wang Y (*n* = 16). Analyzing the publication trends of these authors over the years (Figure 5C) shows that Araya J and Kuwano K have been deeply engaged in COPD autophagy research and gained significant attention over the past decade. The top five most influential authors in this domain were Choi AMK (H-index = 18), Araya J (H-index = 14), Kuwano K (H-index = 14), Rahman I (H-index = 14), and Ryter SW (H-index = 14). Our co-authorship analysis (Figure 5A) demonstrated close collaborations among authors, typically centered around a leading figure. For example, the most influential author Choi AMK has a large collaborative network centered around him, including researchers like Choi Augustine M.K., Ryter Stefan W., and Cloonan Suzanne M. Cocited author co-occurrence analysis (Figure 5B) revealed four clusters where these authors have a strong influence in COPD autophagy research. Based on the visualization, the top three influential authors were Choi AMK (citations = 604), Ryter SW (citations = 555), and Araya J (citations = 354).

**Table 3:** Top 10 authors and cocited authors on the research of autophagy in COPD.

Rank	Authors	Count	Cocited authors	Citations
1	Araya J	19	Choi AMK	604
2	Kuwano K	19	Ryter SW	555
3	Choi AMK	18	Araya J	354
4	Rahman I	16	Kuwano K	354
5	Wang Y	16	Chen ZH	342
6	Ryter SW	14	Kim HP	330
7	Hara H	13	Hara H	329
8	Vij N	13	Ito S	305
9	Yao HW	12	Minagawa S	305
10	Barreiro E	11	Numata T	305

### 3.5 Cocited references

**Figure 6:**
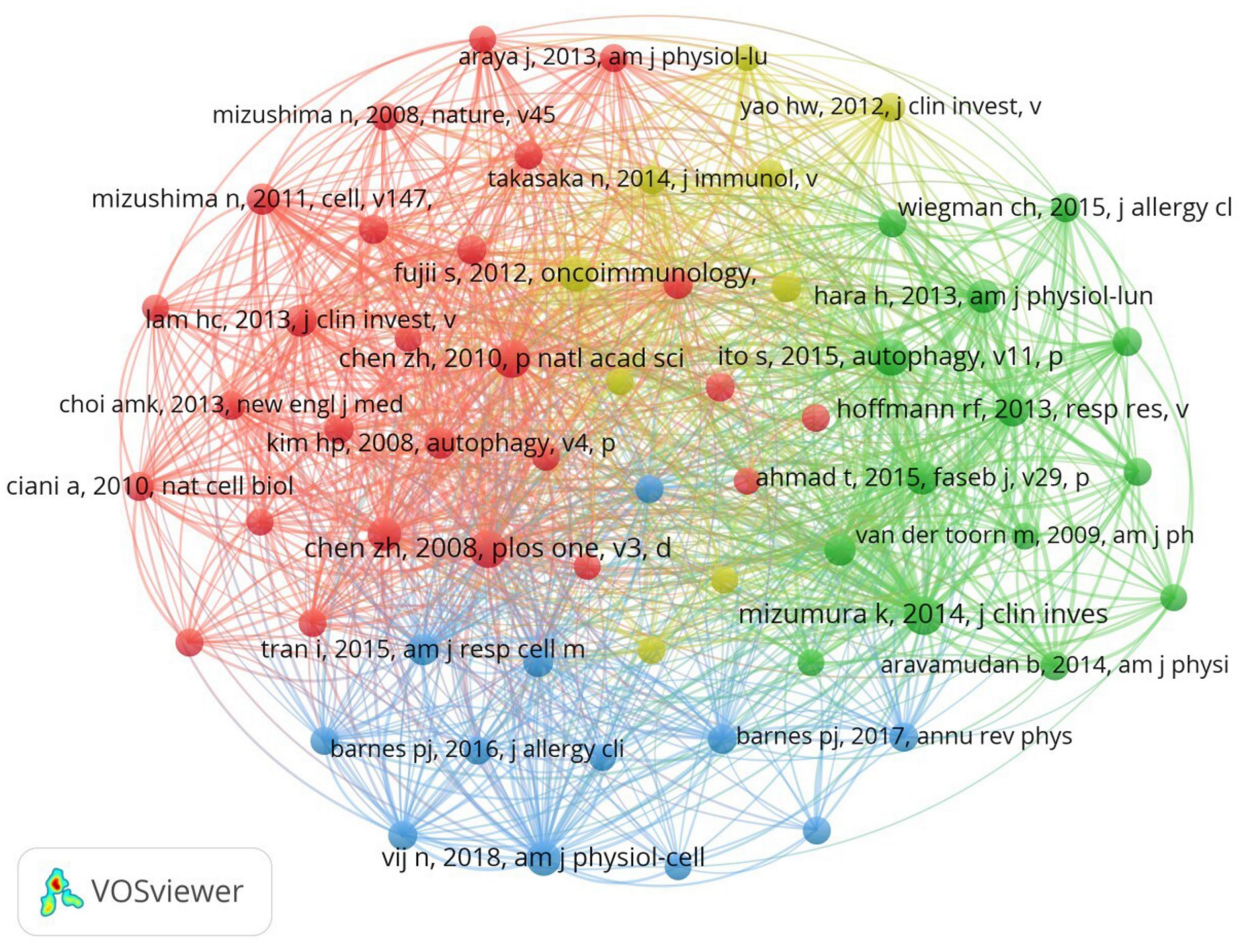
The visualization of cocited references on the research of autophagy in COPD. The node and line color represented the cluster it belonged to COPD.

The past 20 years have seen a substantial volume of cocited references in COPD autophagy research, with an impressive 26,620 references. Among the top 10 most cited references (as shown in Table 4), each garnered no fewer than 50 citations, and three surpassed a remarkable 100 citations. To construct a cocitation network map (Figure 6), we specifically selected references cited 20 times or more. Figure 6 appropriately illustrates how “Mizumura K, 2014, J Clin Invest” shares proactive cocitation relationships with significant references like “Mizumura K, 2014, J Clin Invest,” “Ito S, 2015, Autophagy,” and “Hoffmann RF, 2013, Resp Res.” These findings highlight the significant prevalence of cocited literature around autophagy research in COPD, with particular emphasis on the most frequently cited references that have substantially impacted the field.

**Table 4:** Top 10 cited references on the research of autophagy in COPD.

Rank	Cited references	Citations
1	Mizumura K, 2014, J Clin Invest, V124, P3987, DOI 10.1172/JCI74985	117
2	Chen ZH, 2008, Plos One, V3, DOI 10.1371/journal.pone.0003316	112
3	Chen ZH, 2010, P Natl Acad Sci USA, V107, P18880, DOI 10.1073/pnas.1005574107	103
4	Ito S, 2015, Autophagy, V11, P547, DOI 10.1080/15548627.2015.1017190	94
5	Fujii S, 2012, Oncoimmunology, V1, P630, DOI 10.4161/onci.20297	83
6	Vij N, 2018, AM J Physiol-Cell PH, V314, PC73, DOI 10.1152/Ajpcell.00110.2016	65
7	Hoffmann RF, 2013, Resp Res, V14, DOI 10.1186/1465-9921-14-97	63
8	Monick MM, 2010, J Immunol, V185, P5425, DOI 10.4049/jimmunol.1001603	63
9	Ahmad T, 2015, Faseb J, V29, P2912, DOI 10.1096/FJ.14-268276	59
10	Hara H, 2013, AM J Physiol-Lung C, V305, PL737, DOI 10.1152/ajplung.00146.2013	57

### 3.6 Reference with citation bursts

**Figure 7:**
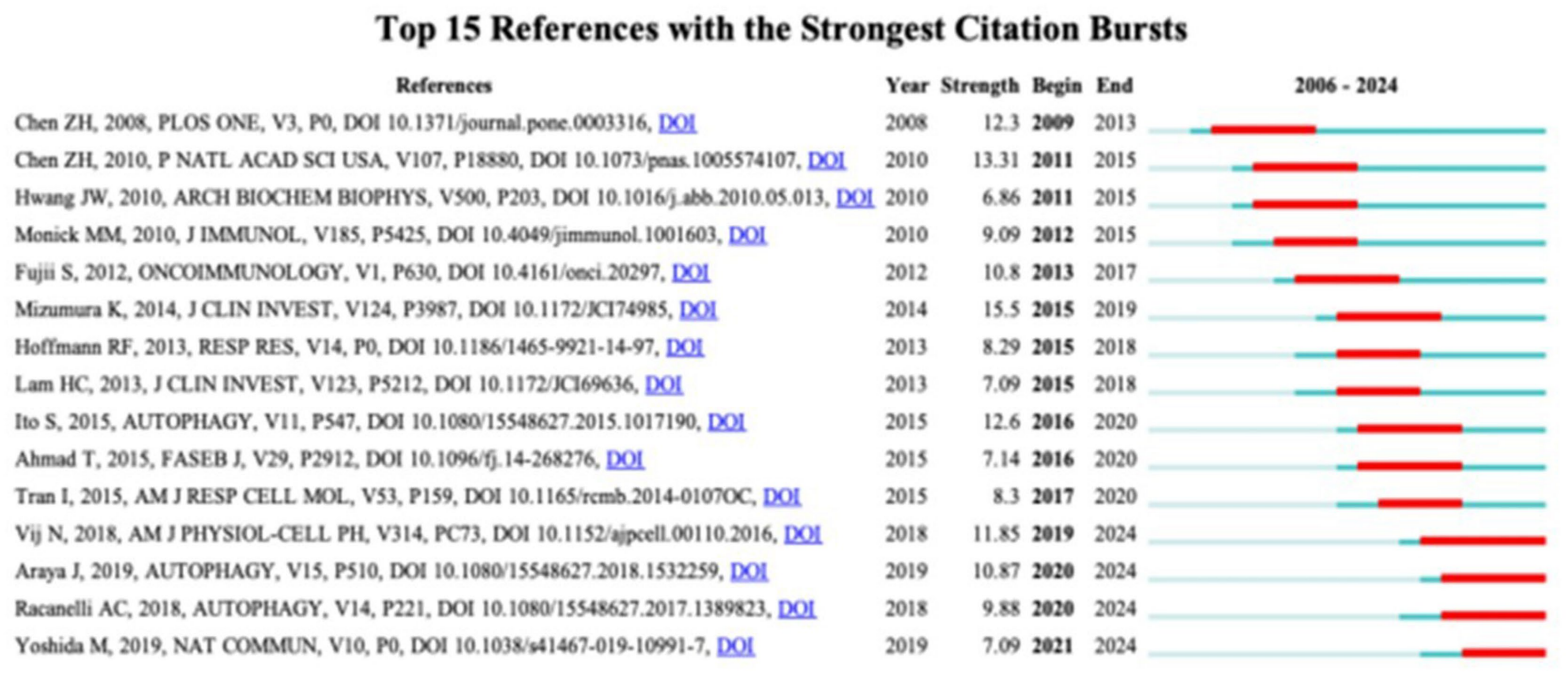
Top 15 references with strong citation bursts. A red bar indicates high citations in that year.

In academic research, references with “citation bursts” indicate periods of intense focus within an extended specific discipline. In our investigation, we utilized CiteSpace to identify and evaluate references showing strong citation bursts, as depicted in Figure 7. This visualization presents notable red bars symbolizing citation bursts, spanning from 2009 to 2024. Among these, a particularly prominent paper is “Cigarette smoke-induced autophagy impairment accelerates lung aging, COPD-emphysema exacerbations and pathogenesis,” by Neeraj Vij et al., with a citation burst from 2019 to 2024. The study titled “Mitophagy-dependent necroptosis contributes to the pathogenesis of COPD,” authored by Kenji Mizumura and published in the prestigious JCI journal, has the highest initial citation burst strength. The citation burst strength for these 15 references varies from 6.86 to 15.5, lasting 3–5 years. Table 5 provides a comprehensive overview of the main research themes of these 15 references, corresponding to their respective positions in Figure 7. This analysis offers valuable insights into the academic landscape surrounding these influential references in our research area.

**Table 5:** The main research contents of the 15 references with strong citations bursts.

Rank	Strength	Main research content
1	12.3029	In COPD patients, increased autophagy activity in lung tissue is driven by Egr-1 in response to cigarette smoke, suggesting that Egr-1 may serve as a novel therapeutic target for treating cigarette-induced lung injury.
2	13.3101	This study elucidates the pivotal role of the autophagic protein LC3B in mediating cigarette smoke-induced apoptosis and emphysema in chronic obstructive pulmonary disease (COPD), revealing its interaction with caveolin-1 and Fas, and suggesting novel therapeutic targets for COPD treatment.
3	6.8568	This study investigates the critical role of the SIRT1–PARP-1 axis in regulating cigarette smoke-induced autophagy in lung cells, revealing that SIRT1 activation modulates autophagy responses to oxidative stress from cigarette smoke and provides insights into mechanisms of cell death and senescence.
4	9.0937	This study reveals that smokers’ alveolar macrophages exhibit an autophagy defect characterized by the accumulation of autophagosomes and p62, leading to impaired bacterial clearance and increased infection susceptibility, suggesting that targeting the autophagy pathway may help reduce infection rates in smokers and those exposed to high environmental particulate matter.
5	10.8026	This study demonstrates that tobacco smoke-induced autophagy activation in primary human bronchial epithelial cells is transient and leads to accelerated cell senescence, characterized by the accumulation of p62 and ubiquitinated proteins, highlighting that insufficient autophagic clearance contributes to cell senescence in chronic obstructive pulmonary disease (COPD) and suggesting a potential protective role for autophagy against tobacco smoke-induced lung damage.
6	15.502	This study identifies mitophagy-dependent necroptosis as a key mechanism in cigarette smoke-induced lung damage and emphysema in chronic obstructive pulmonary disease (COPD), demonstrating that mitochondrial dysfunction and PINK1 stabilization play critical roles, thus suggesting this pathway as a potential therapeutic target for COPD.
7	8.2891	This study demonstrates that long-term exposure to cigarette smoke induces significant structural and functional changes in airway epithelial mitochondria, characterized by fragmentation and alterations in key mitochondrial markers, which are associated with increased oxidative stress and pro-inflammatory mediators, suggesting that these mitochondrial impairments may contribute to the pathogenesis of chronic obstructive pulmonary disease (COPD).
8	7.0944	This study reveals that autophagy plays a crucial role in regulating cilia length and mucociliary clearance disruption in chronic obstructive pulmonary disease (COPD) due to cigarette smoke exposure, identifying HDAC6 as a key regulator of this autophagy-mediated process, and suggesting that targeting this pathway may offer therapeutic benefits for COPD.
9	12.5969	This study investigates the role of PINK1-PARK2-mediated mitophagy in regulating cigarette smoke extract-induced mitochondrial damage and cellular senescence in primary human bronchial epithelial cells, revealing that reduced PARK2 expression in COPD lungs contributes to insufficient mitophagy and enhanced oxidative stress, implicating this pathway in COPD pathogenesis.
10	7.1357	This study reveals that cigarette smoke-induced cellular senescence in chronic obstructive pulmonary disease (COPD) is linked to impaired mitophagy due to reduced Parkin translocation, driven by cytoplasmic p53 accumulation, and demonstrates that restoring mitophagy can delay senescence, suggesting a potential therapeutic approach for chronic airway diseases.
11	8.3039	This study identifies cigarette smoke-induced aggresome formation, characterized by the accumulation of ubiquitinated proteins in perinuclear spaces, as a novel mechanism contributing to chronic obstructive pulmonary disease (COPD) and emphysema pathogenesis, highlighting impaired autophagy and increased p62 accumulation as key factors in this process.
12	11.8471	This study investigates how cigarette smoke exposure and aging contribute to chronic obstructive pulmonary disease (COPD) and emphysema through impaired autophagy and the accumulation of aggresome bodies, demonstrating that autophagy induction via the antioxidant cysteamine can mitigate these effects and reduce pulmonary exacerbations related to bacterial infections.
13	10.8714	This study clarifies the role of PRKN-regulated mitophagy in chronic obstructive pulmonary disease (COPD) pathogenesis, demonstrating that PRKN knockout mice exhibit exacerbated airway damage and cellular senescence following cigarette smoke exposure, while PRKN overexpression can induce mitophagy and reduce oxidative stress, indicating that PRKN levels may be crucial for mitigating COPD progression.
14	9.8774	This perspective discusses the dual role of autophagy in lung inflammation, emphasizing its critical function in regulating inflammatory responses to infections and stress in chronic pulmonary diseases, while highlighting the potential for therapeutic targeting to address persistent inflammation that can lead to lung injury.
15	7.0927	This study reveals that ferroptosis, characterized by phospholipid peroxidation and iron accumulation, plays a significant role in chronic obstructive pulmonary disease (COPD) pathogenesis, as demonstrated by increased lipid peroxidation and non-apoptotic cell death in cigarette smoke-exposed lung epithelial cells, with GPx4 regulation being crucial in this process.

### 3.7 Hotspots and frontiers

**Figure 8:**
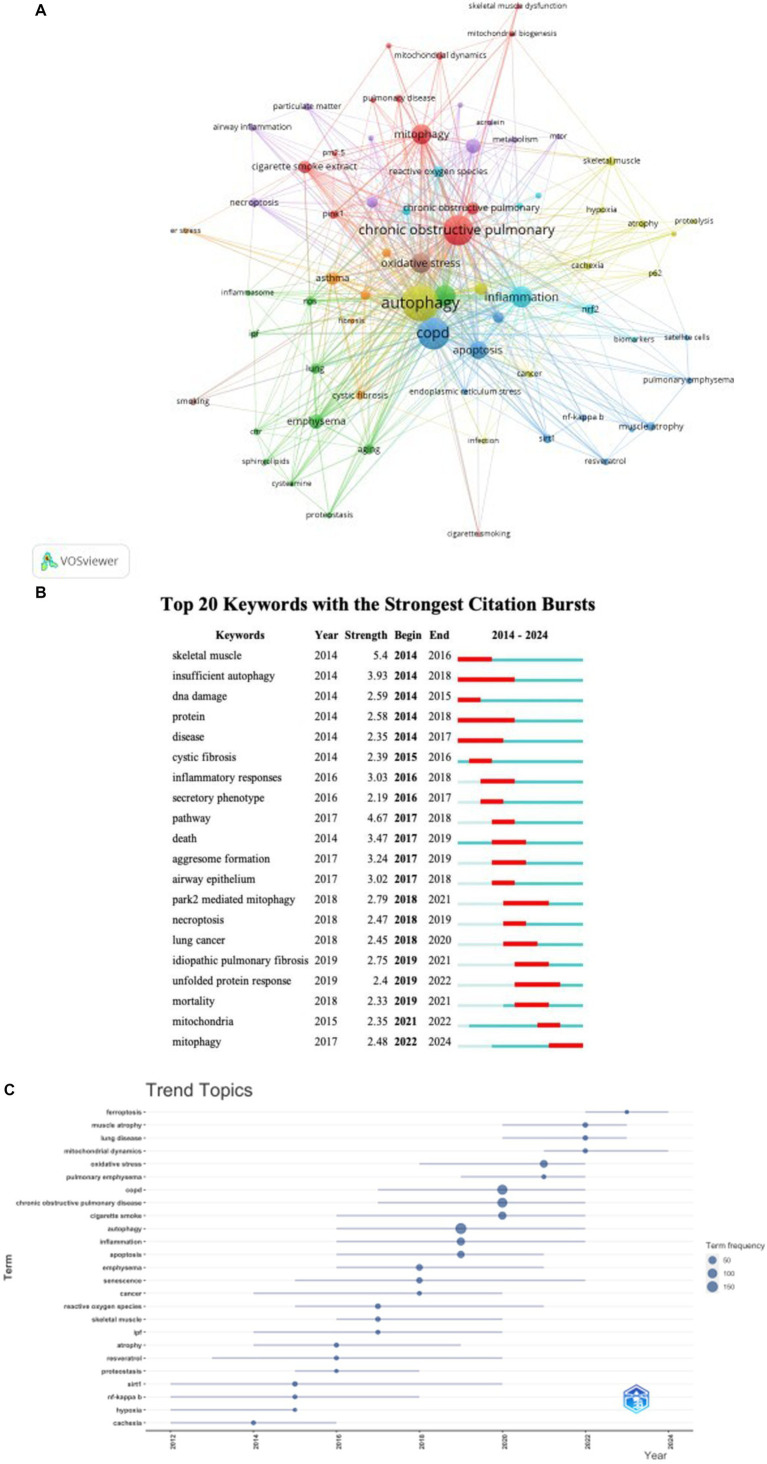
Keyword cluster analysis **(A)** and trend topic analysis **(C)**. In A, the node and line color represented the cluster it belonged to COPD. Top 20 keywords **(B)** with strong citation bursts. A red bar indicates high citations in that year.

Keyword co-occurrence analysis is a valuable tool for quickly identifying research focuses within a specific field. In our research on autophagy in COPD, Table 6 lists 20 high-frequency keywords (as shown in Figure 8B). Notably, “oxidative stress,” “apoptosis,” “senescence,” and “inflammation” prominently appeared, each with over 40 instances, indicating their central role in COPD autophagy research. To derive meaningful insights, we filtered keywords with occurrences of 5 or more and conducted clustering analysis using VOSviewer (as shown in Figure 8A). The width of lines connecting nodes in the map depicts the level of linkage strength between different keywords. Figure 8A reveals three distinct clusters, each representing a unique research direction. The yellow cluster includes keywords such as “autophagy,” “atrophy,” “hypoxia,” “skeletal muscle,” “cachexia,” and “p62.” The red cluster encompasses keywords related to “chronic obstructive pulmonary disease,” “mitophagy,” “pink1,” and “mitochondrial dynamics.” Meanwhile, the blue cluster contains keywords associated with “apoptosis,” “endoplasmic reticulum stress,” “sirt1,” “NF-κB,” and related concepts. As shown in Figure 8C, the trend analysis of keywords indicates that between 2012 and 2016, research mainly focused on the regulatory roles of upstream molecular signaling pathways like sirt1 and NF-κB in autophagy. Dominant terms of this era included “sirt1,” “NF-κB,” “hypoxia,” “cachexia,” and “resveratrol.” This period witnessed significant efforts in the biochemical aspects of COPD, particularly regarding resveratrol, sirt1, and NF-κB. From 2016 to 2020, scholars actively explored the micro-molecular mechanisms of autophagy in COPD, especially atrophy. Key terms of this era included “inflammation,” “autophagy,” “emphysema,” “senescence,” “reactive oxygen species,” and “skeletal muscle.” This stage highlights the significant complexity of mechanisms revealed within COPD’s context. Subsequently, since 2020, scholars have shifted focus to studying micro-molecular mechanisms controlling mitochondria in COPD. At this stage, central keywords include “oxidative stress,” “mitochondrial dynamics,” “ferroptosis,” “muscle atrophy,” and related concepts. This shift indicates heightened interest in understanding finer molecular details of mitochondria involvement in COPD. Furthermore, the last 4 years (2020–2024) saw a marked increase in interest around four pivotal keywords. These keywords, notably “ferroptosis,” “muscle atrophy,” “lung disease,” and “mitochondrial dynamics,” have garnered considerable attention. Their frequent appearance in current studies suggests they may represent major research hotspots in the COPD mitochondria field. These emerging themes indicate evolving trends and research focuses within this dynamic research domain.

**Table 6:** Top 20 high-frequency keywords.

Rank	Keywords	Count	Rank	Keywords	Count
1	autophagy	142	11	cigarette-smoke	47
2	oxidative stress	126	12	senescence	43
3	obstructive pulmonary-disease	117	13	mechanisms	42
4	copd	101	14	cells	41
5	expression	77	15	disease	38
6	inflammation	76	16	nf-kappa-b	38
7	apoptosis	73	17	dysfunction	37
8	pathogenesis	54	18	emphysema	37
9	mitophagy	52	19	lung	32
10	activation	48	20	cell-death	27

## 4 Discussion

Chronic obstructive pulmonary disease (COPD) is a progressive respiratory condition characterized by airflow limitation and chronic inflammation, affecting millions of people worldwide (9). Recent studies have highlighted the importance of autophagy in the pathogenesis of COPD (17). Dysregulated autophagy has been linked to chronic inflammation (18), airway remodeling (19), and cellular stress (20). In COPD, dysregulated autophagy contributes to chronic inflammation, tissue damage, and reduced lung function. While autophagy is initially upregulated in response to stressors like cigarette smoke, it can become overwhelmed, leading to an accumulation of dysfunctional components (21). Infections from bacteria and viruses significantly affect autophagy in lung cells, with higher bacterial loads correlating with increased bronchial inflammation and production of pro-inflammatory cytokines such as IL-1β and TNF-*α*, which exacerbates inflammation and can trigger acute exacerbations (22). The COVID-19 pandemic has complicated this further, as SARS-CoV-2 manipulates autophagy for replication while causing autophagic dysregulation, leading to increased inflammation in COPD patients (23). Additionally, diseases like cancer and pulmonary hypertension interact with autophagy; tumor cells may exploit autophagy to evade apoptosis, impacting treatment effectiveness (24), while pulmonary hypertension is linked to impaired autophagy, worsening respiratory function (25).

### 4.1 General information

In this study, we utilized bibliometric analysis tools such as R, VOSviewer, and CiteSpace to map research trends and key contributors in the field of autophagy and COPD. Our findings provide a comprehensive overview.

The bibliometric analysis reveals a general increase in the number of publications on autophagy and COPD over the past 20 years, totaling 481 journal articles across 229 publications. The first article appeared in 2006, suggesting that excessive autophagy might contribute to muscle dysfunction in COPD patients (26). While the field saw significant growth, particularly in the last decade, a decline in publications after 2022 indicates a decreased focus on this area. This shift highlights the need to reassess research priorities to better understand autophagy’s role in COPD progression and therapeutic strategies.

The country-specific publication data indicates that China ranks first in the number of publications on autophagy and COPD, followed closely by the United States. Other countries, such as Japan and the Netherlands, have published significantly fewer articles compared to these two. This discrepancy may be associated with the higher prevalence of COPD and the focus on chronic disease management in China and the U.S. Despite leading in publication volume, China’s international collaboration in this field is notably lower than that of other nations, highlighting the need for enhanced global cooperation in future research.

Among the top 10 institutions in terms of publication volume, six are from the United States, and three are from China. Interestingly, Maastricht University ranks highest in NP (number of publications) and has the most published articles. The institutional ranking highlights that the leading contributors to research on autophagy and COPD are primarily from the U.S. and China. However, compared to institutions in the U.S. and Japan, Chinese institutions tend to receive fewer citations, suggesting that while they are making significant contributions, their overall impact in terms of citation influence is relatively lower. Enhancing research quality and visibility may help increase the global recognition and impact of Chinese institutions in future studies.

In our bibliometric analysis of publications related to autophagy and COPD, three journals stand out in terms of both publication volume and citation frequency: American Journal of Physiology - Lung Cellular and Molecular Physiology (AM J PHYSIOL-LUNG C), International Journal of Chronic Obstructive Pulmonary Disease and Autophagy (INT J CHRONIC OBSTR). INT J CHRONIC OBSTR is a leading journal in respiratory medicine, well-known for publishing high-quality clinical and basic research, as well as comprehensive reviews in the field. This journal plays a pivotal role in disseminating key findings that drive advancements in the diagnosis and treatment of respiratory diseases, including COPD. AM J PHYSIOL-LUNG C, on the other hand, focuses primarily on molecular and cellular mechanisms governing both normal and pathological functions of the respiratory system. It serves as a platform for foundational research that furthers our understanding of lung biology. Lastly, Autophagy is the authoritative journal in its field, offering in-depth explorations of the autophagic process and its implications in various diseases, including COPD. These journals are essential for disseminating critical discoveries at the intersection of autophagy and COPD.

Our research shows that studies on autophagy in COPD began relatively late. In 2009, Ryter SW et al. conducted a groundbreaking study, revealing that autophagy plays a vital role in maintaining cellular balance, particularly during nutrient deprivation (27). Although autophagy is widely recognized as a survival mechanism, its links to cell death pathways remain unclear and had not previously been explored in the context of lung diseases. Their study identified elevated autophagy markers in lung tissues from COPD patients and in mice exposed to cigarette smoke, a major COPD trigger. Similar results were observed in pulmonary cells treated with cigarette smoke extract (28, 29). They concluded that increased autophagy was linked to higher cell death in response to smoke exposure, as reducing autophagy-related proteins decreased apoptosis. This suggests that autophagy may contribute to COPD progression by promoting epithelial cell death.

In 2011, Kim HP et al. found that cigarette smoke extract (CSE) plays a critical role in COPD pathogenesis by inducing both autophagy and apoptosis in bronchial epithelial cells. CSE increased autophagosome formation and LC3B conversion, confirming elevated autophagy. Concurrently, CSE triggered extrinsic apoptosis through DISC formation and caspase activation, both dependent on autophagic proteins. Notably, HO-1 downregulated these pathways, protecting cells from smoke-induced death by reducing DISC formation, caspase activation, and autophagy. HO-1 also modulated NF-κB signaling, further enhancing cell survival (30). Published in 2011, another study revealed that autophagy markers were elevated in the lungs of COPD patients and mice exposed to cigarette smoke. LC3B, a key autophagic protein, was shown to promote lung cell apoptosis and tissue damage during smoke exposure. In LC3B knockout mice, lung apoptosis was reduced, and airspace enlargement was mitigated. LC3B interacts with the death receptor Fas in epithelial cells, a process dependent on caveolin-1. Notably, caveolin-1 knockout mice exhibited increased autophagy and apoptosis, suggesting that targeting the autophagic pathway may offer a promising therapeutic strategy for COPD treatment (31). A 2012 study found that CSE transiently activates autophagy in bronchial epithelial cells, followed by accelerated cell aging and accumulation of p62 and ubiquitinated proteins. Inhibiting autophagy increased protein accumulation, senescence, and secretion of inflammatory cytokines like IL-8. Conversely, autophagy activation reduced these effects. In COPD patients, autophagy response to CSE was impaired, leading to more protein buildup and cell senescence (32). A 2013 clinical study revealed that autophagy is markedly increased in the locomotor muscles of COPD patients, contributing to muscle atrophy. Higher levels of autophagosomes, LC3B lipidation, and autophagy-related gene expression were observed, correlating with impaired lung function. Autophagy activation was associated with the AMPK and FOXO pathways (33).

After establishing the link between macroautophagy and COPD, researchers shifted their attention to selective autophagy, especially mitophagy (34, 35), and the upstream and downstream mechanisms of autophagy (36, 37). A 2014 study showed that cigarette smoke (CS) causes mitochondrial dysfunction and reduces membrane potential, triggering mitophagy through PINK1 stabilization. Inhibiting necroptosis reduced CS-induced cell death and mitochondrial damage. PINK1 deficiency and Mdivi-1, a mitophagy inhibitor, protected against CS-induced damage, reducing MLKL phosphorylation. Pink1(−/−) mice showed protection against mitochondrial dysfunction, airspace enlargement, and mucociliary clearance disruption (38). In a 2019 study, Araya and colleagues explored PRKN-regulated mitophagy in COPD using prkn knockout mice (39). After cigarette smoke exposure, these mice showed increased airway wall thickening, emphysematous changes, mitochondrial damage, and accelerated cell senescence. PRKN overexpression in airway epithelial cells promoted mitophagy, reducing mitochondrial ROS and senescence, even with low PINK1 levels. However, PINK1 overexpression could not compensate for PRKN knockdown, indicating PRKN as the key regulator in PINK1-PRKN-mediated mitophagy during cigarette smoke exposure. A 2016 study explored the upstream and downstream mechanisms of autophagy in COPD. Researchers found that PM2.5 exposure in bronchial epithelial cells led to increased VEGFA production, which is involved in airway inflammation and vascular remodeling. Autophagy was activated upon PM2.5 exposure, mediating VEGFA upregulation via the SRC-STAT3 pathway. Further investigations revealed that TP53 activation and its downstream target DRAM1 were necessary for autophagy induction. Additionally, the ATR-CHEK1 axis was shown to activate TP53-dependent autophagy and VEGFA production in response to PM2.5 exposure, highlighting a role for autophagy in controlling proinflammatory cytokine production (19).

In summary, recent studies strongly suggest a close relationship between autophagy and COPD. Researchers have made valuable discoveries regarding autophagy and its upstream and downstream mechanisms, demonstrating the potential of autophagy as a therapeutic target for COPD.

### 4.2 Hotspots and frontiers

Keywords play a crucial role in bibliometric studies, allowing for the rapid identification of research hotspots and trends in the evolution of autophagy studies. By analyzing keywords, we can effectively map out the distribution of research focus and track how the field has developed over time.

Beyond specific terms like “autophagy,” “obstructive pulmonary disease,” and “COPD,” other major keywords include “oxidative stress,” “inflammation,” “apoptosis,” “senescence,” and “NF-kappa-B.” These terms are essential as they represent key mechanisms involved in COPD pathogenesis. Oxidative stress and inflammation are central to COPD’s progression

while apoptosis and senescence highlight cell death and aging processes. NF-kappa-B is a critical regulator of inflammation and immune response (40, 41).

Through keyword cluster analysis and trend topic analysis, we identified that the main research focus of autophagy in COPD is concentrated on oxidative stress, inflammation, apoptosis, and senescence. These interconnected pathways play significant roles in understanding the disease’s molecular mechanisms, paving the way for potential therapeutic strategies targeting autophagy.

#### 4.2.1 Oxidative stress

Oxidative stress is a critical factor in the pathogenesis of COPD and plays a significant role in regulating autophagy in this disease (42). Oxidative stress occurs when there is an imbalance between the production of reactive oxygen species (ROS) and the ability to neutralize them with antioxidants. In COPD, chronic exposure to environmental pollutants, particularly cigarette smoke, leads to excessive ROS generation in the lungs, resulting in oxidative damage to cellular structures, including lipids, proteins, and DNA (43, 44).

In COPD, however, this protective mechanism often becomes impaired. Studies have shown that oxidative stress can dysregulate autophagy, leading to either excessive or insufficient autophagic activity (45). Excessive ROS production can trigger overactivation of autophagy, which may contribute to cell death via autophagic mechanisms. Onversely, chronic oxidative stress can lead to defective autophagic flux, where the autophagosomes that form in response to cellular damage fail to fuse with lysosomes (46), preventing the proper degradation of damaged organelles and proteins. This impaired autophagy contributes to the accumulation of cellular debris and perpetuates oxidative damage, further aggravating the progression of COPD (47).

One of the key signaling pathways that links oxidative stress to autophagy regulation is the mTOR (mechanistic target of rapamycin) pathway. Under oxidative stress conditions, the activation of AMPK (AMP-activated protein kinase) inhibits mTOR (48), thereby promoting autophagy. In COPD, chronic oxidative stress can alter the balance between AMPK and mTOR, leading to either excessive or insufficient autophagic responses, depending on the context and severity of oxidative damage (49). This dysregulation of the AMPK-mTOR axis is a critical aspect of how oxidative stress modulates autophagy in COPD.

Moreover, oxidative stress can directly influence key regulators of autophagy, such as the transcription factor TFEB (Transcription Factor EB), which controls the expression of autophagy-related genes. Under high oxidative stress, TFEB may be dysregulated, leading to impaired autophagy (50). Additionally, ROS can activate redox-sensitive transcription factors, such as NF-kappa-B, which not only drives inflammatory responses but also impacts autophagy. Chronic activation of NF-kappa-B in response to oxidative stress may lead to a sustained inflammatory response that overwhelms the autophagic machinery, contributing to lung tissue damage in COPD (51).

Interestingly, autophagy can also have a feedback effect on oxidative stress. Proper autophagic activity helps limit oxidative stress by clearing ROS-producing organelles (43), whereas defective autophagy can exacerbate oxidative stress by allowing damaged mitochondria and other cellular components to persist (52). This feedback loop highlights the intricate relationship between oxidative stress and autophagy in COPD pathogenesis.

In summary, oxidative stress is a central driver of autophagy dysregulation in COPD. While autophagy initially serves to mitigate oxidative damage by removing damaged organelles and proteins, chronic oxidative stress can impair autophagic function, leading to either excessive autophagy-mediated cell death or insufficient clearance of damaged components. Understanding the balance between oxidative stress and autophagy regulation is crucial for identifying therapeutic targets that can restore proper autophagic function and alleviate oxidative damage in COPD.

#### 4.2.2 Inflammation

Inflammation is a fundamental process in COPD pathogenesis, and its interplay with autophagy is increasingly recognized as a key mechanism driving disease progression. In COPD, chronic inflammatory responses are triggered by environmental factors, particularly cigarette smoke, leading to the sustained activation of immune cells such as neutrophils and macrophages (53). These immune cells release a cascade of pro-inflammatory mediators, including TNF-*α*, IL-6, IL-1β, and IL-8, which promote tissue injury, remodeling, and progressive loss of lung function (54).

Autophagy, a crucial homeostatic process, is responsible for the degradation and recycling of damaged organelles and proteins. It plays a dual role in regulating inflammatory responses (55). In the context of COPD, autophagy has been shown to control excessive inflammation by degrading damaged mitochondria (56), thus preventing the release of mitochondrial DNA (mtDNA) and reactive oxygen species (ROS) that could otherwise act as damage-associated molecular patterns (DAMPs) to further amplify inflammatory signaling. This protective role of autophagy is essential for limiting chronic inflammation and preventing exacerbation of COPD symptoms.

However, evidence suggests that impaired or dysregulated autophagy may exacerbate inflammation in COPD. Chronic exposure to cigarette smoke and other toxicants can lead to defective autophagic flux, characterized by the accumulation of autophagosomes that fail to fuse with lysosomes (57). This results in the incomplete degradation of cellular debris and damaged mitochondria, leading to an increase in ROS production. ROS are potent inflammatory signals that can activate key pathways such as PARK2, is involved in the regulation of mitochondrial homeostasis, energy metabolism and other cellular processes. Mitophagy was inhibited by PARK2 knockdown, resulting in enhanced mitochondrial ROS production and cellular senescence in primary human bronchial epithelial cells (58).

In addition to ROS-mediated inflammation, other studies have shown that autophagy can modulate the NLRP3 inflammasome, a multiprotein complex that activates inflammatory cytokines like IL-1β and IL-18 (59). In COPD, defective autophagy may impair the clearance of inflammasome components, leading to their prolonged activation and further aggravating inflammation. This highlights the complex, bidirectional relationship between autophagy and inflammatory signaling (60).

Furthermore, specific molecular regulators of autophagy, such as the AMPK and mTOR pathways, are directly linked to inflammatory responses. AMPK activation promotes autophagy and has anti-inflammatory effects (61), while mTOR inhibition, often associated with enhanced autophagy, has been shown to reduce inflammation (62). These pathways represent potential therapeutic targets, as modulating autophagy through these mechanisms could help dampen the chronic inflammation characteristic of COPD.

In summary, inflammation and autophagy are intricately connected in the pathogenesis of COPD. While autophagy can act as a protective mechanism by controlling inflammation and removing damaged cellular components, its dysregulation may intensify inflammatory responses, promoting further lung damage and disease progression. Understanding the precise mechanisms by which autophagy influences inflammation in COPD is crucial for developing targeted therapies that could restore autophagic function and mitigate chronic inflammation in COPD patients.

#### 4.2.3 Apoptosis

In COPD, the interplay between apoptosis and autophagy is a critical and complex process. Apoptosis, a form of programmed cell death, is prominent in the lung tissues of COPD patients, particularly following exposure to cigarette smoke and environmental toxins (63, 64). Autophagy and apoptosis are often in a regulatory balance; however, this balance is disrupted in COPD, leading to excessive cell death and exacerbating disease progression.

Autophagy can protect against apoptosis by removing damaged organelles, such as mitochondria, which are sources of reactive oxygen species (ROS). Mitophagy, the selective degradation of damaged mitochondria, helps reduce ROS accumulation, thereby inhibiting apoptosis (65, 66). In COPD, however, impaired autophagy fails to clear damaged mitochondria effectively, resulting in increased ROS levels, which in turn trigger apoptotic pathways (67), including caspase activation and the formation of death-inducing signaling complexes (DISC), promoting further cell death (30).

Additionally, autophagy-related proteins like Beclin-1 and LC3B are involved in apoptosis regulation. Beclin-1, a key autophagy regulator, interacts with the anti-apoptotic protein Bcl-2 (68, 69). Disruption of this interaction can shift the balance toward increased apoptosis. In COPD, stressors such as cigarette smoke can modulate these proteins, further amplifying cell death and tissue injury.

The p53 pathway also plays a significant role in this process. Under stress, p53 can activate both autophagy and apoptosis via mitochondrial pathways (70). In COPD, p53 overactivation is associated with increased apoptosis and autophagy dysfunction (19), indicating that p53 serves as a critical link between these two processes.

Moreover, the imbalance between apoptosis and autophagy may contribute to tissue fibrosis and structural damage in COPD. Excessive apoptosis leads to the loss of epithelial cells, disrupting alveolar structures (47), while defective autophagy hampers the clearance of damaged cells. Together, these mechanisms accelerate lung tissue degradation and airway remodeling, further driving COPD progression.

#### 4.2.4 Senescence

Cellular senescence, the permanent cessation of cell division, is a critical process contributing to the pathology of COPD. Unlike apoptosis, which leads to programmed cell death, senescent cells remain metabolically active and can persist in lung tissue, driving chronic inflammation and tissue dysfunction (45). In COPD, environmental stressors such as cigarette smoke accelerate senescence (71), particularly in bronchial epithelial cells, fibroblasts, and alveolar macrophages. This accumulation of senescent cells is associated with impaired lung repair and sustained inflammatory responses.

Senescent cells, once established, promote inflammation through the senescence-associated secretory phenotype (SASP), which involves the release of cytokines, chemokines, and matrix metalloproteinases (MMPs) (72). These factors not only propagate inflammation but also cause tissue remodeling and further lung damage. In pulmonary disease, the inability of autophagy to eliminate senescent cells allows them to persist in the lung environment, where they continue to secrete SASP factors, maintaining a chronic inflammatory state (73). This contributes to progressive alveolar destruction, airway remodeling, and impaired lung function.

Additionally, senescence and autophagy are co-regulated by several molecular pathways, including SIRT1 and mTOR signaling. SIRT1 is an NAD (+) dependent deacetylase that is involved in a wide variety of biological processes, such as metabolism, immune response, and aging. During senescence, nuclear SIRT1 is identified as a substrate for autophagy and undergoes degradation in the cytoplasm through the autophagosome-lysosome pathway, mediated by the autophagy-related protein LC3. In smokers and individuals with COPD, SIRT1 levels were decreased in macrophages and lung tissues due to post-translational modifications caused by reactive components from cigarette smoke (74).

Another key aspect of senescence in COPD is its impact on tissue repair. Senescent cells, unlike apoptotic cells, are not efficiently cleared through normal cellular mechanisms, leading to their accumulation over time. This impairs the lung’s ability to repair damaged tissue, contributing to the progressive nature of COPD (75, 76). The chronic presence of senescent cells exacerbates tissue fibrosis and emphysema, making the disease more difficult to manage as it advances.

### 4.3 Advantages and limitations

This study systematically reveals the trends, key research institutions, and collaborations between countries in the field of autophagy and chronic obstructive pulmonary disease (COPD) through bibliometric analysis, providing deep insights into this research area. On one hand, this analysis utilizes various tools such as R, VOSviewer, and CiteSpace to reveal research dynamics and impacts from different perspectives. This provides valuable foundational data for researchers to identify core themes and guide future research directions.

However, the study also has some limitations. Firstly, it relies solely on the Web of Science Core Collection (WoSCC) database for literature screening, which may lead to the omission of relevant research results contained in other databases or gray literature. Additionally, since only English literature was analyzed, the contributions and impacts of non-English publications may not be fully reflected.

An important point is that the bibliometric study has showcased the rich research output from several major academic centers worldwide, particularly those located in developed countries and large nations like China. However, according to the latest data from the GBD database, East Asia, South Asia, and Southeast Asia have the highest number of deaths and DALYs attributable to COPD. South Asia exhibits the highest age-standardized prevalence rates for COPD, exceeding 3,000 cases per 100,000 people. In terms of mortality and DALYs, the age-standardized rates in Oceania, South Asia, and East Asia are all above the global average (77). These areas face significant COPD prevalence due to widespread tobacco use, exposure to biomass fuel, and a lack of healthcare resources. Bibliometrics primarily relies on existing published literature, which means studies that have not been published or cited may be overlooked, especially in resource-poor countries that lack international collaboration. As a result, bibliometric analysis may fail to accurately reflect the true conditions and research needs of COPD in these high-burden regions.

In light of these limitations, future research should consider the comprehensive use of multiple methodologies to gain a more thorough understanding of the regional characteristics and research needs related to COPD. For instance, integrating the GBD database with local epidemiological data could help reveal the gaps in COPD research within high-burden areas. Additionally, enhancing qualitative research efforts, such as local health surveys and community participatory studies, will provide deeper insights into the environmental and social factors affecting COPD patients, thereby more accurately reflecting the actual healthcare needs in these regions.

It is also crucial to strengthen collaborations with research institutions in these high-burden areas and facilitate the publication of local studies, which will not only enhance research output in these regions but also provide richer perspectives for global COPD research. Moreover, funding support and resource sharing should be prioritized to promote cross-national research collaboration, ensuring the sustainability and applicability of COPD studies.

## 5 Conclusion

In the bibliometric analysis of the intersection of autophagy and COPD, this study highlights the importance of researching autophagic processes in COPD and the international research landscape within the field. The results show that research in this area is mainly concentrated in the United States and China. Although China holds an advantage in terms of publication volume, there is still room for improvement in international collaboration and citation impact. This suggests that there is a need to strengthen international cooperation in the future and focus on enhancing research quality and global influence. By conducting an in-depth analysis of research topics and trends, this study provides important guidance for exploring innovative research directions and developing effective therapeutic strategies. Researchers can utilize these findings to further focus on emerging areas, deepen collaboration, and accelerate breakthroughs in the study of autophagy mechanisms related to COPD.
